# A Computational Reverse Vaccinology Approach for the Design and Development of Multi-Epitopic Vaccine Against Avian Pathogen *Mycoplasma gallisepticum*

**DOI:** 10.3389/fvets.2021.721061

**Published:** 2021-10-26

**Authors:** Susithra Priyadarshni Mugunthan, Harish Mani Chandra

**Affiliations:** Plant Genetic Engineering and Molecular Farming Lab, Department of Biotechnology, Thiruvalluvar University, Vellore, India

**Keywords:** immunoinformatics, *Mycoplasma gallisepticum*, multi-epitopic vaccine, chronic respiratory disease, poultry vaccine

## Abstract

Avian mycoplasma is a bacterial disease causing chronic respiratory disease (CRD) in poultry industries with high economic losses. The eradication of this disease still remains as a challenge. A multi-epitope prophylactic vaccine aiming the antigenic proteins of *Mycoplasma gallisepticum* can be a capable candidate to eradicate this infection. The present study is focused to design a multi-epitope vaccine candidate consisting of cytotoxic T-cell *(*CTL), helper T-cell (HTL), and B-cell epitopes of antigenic proteins, using immunoinformatics strategies. The multi-epitopic vaccine was designed, and its tertiary model was predcited, which was further refined and validated by computational tools. After initial validation, molecular docking was performed between multi-epitope vaccine construct and chicken TLR-2 and 5 receptors, which predicted effective binding. The *in silico* results specify the structural stability, precise specificity, and immunogenic response of the designed multi-epitope vaccine, and it could be an appropriate vaccine candidate for the *M. gallisepticum* infection.

## Introduction

*Mycoplasma gallisepticum* is cell-wall-less bacteria, the key avian respiratory pathogen which is the causative agent for chronic respiratory disease (CRD) in chickens and infectious sinusitis in turkeys and house finches (1). *M. gallisepticum* infection tends to intensify the pathology of accompanying viral infections like Newcastle disease virus (NDV), infectious bronchitis (IB), and CRD in chickens. *M. gallisepticum* is the crucial pathogen in poultry industries leading to significant economic loss by infecting layers and breeder and broiler poultry flocks (2). The reason for CRD outbreak and spread includes heat/cold stress, excessive ammonia, and unhygienic conditions (3, 4). The affected chickens manifest clinical symptoms like respiratory distress, nasal and ocular discharge loss of appetite, and decreased egg production.

*M. gallisepticum* are classified under the class Mollicutes and family *Mycoplasmataceae. M. gallisepticum* has a small genome with 996 kb responsible for its limited biosynthetic capabilities and low replication rates and tends to infect the respiratory tract and colonize in the mucosal surface tract of chicken. Transmission of *M. gallisepticum* in respiratory tract mucosa induces local inflammation in the trachea, lungs, and air sacs and sporadically in the conjunctiva and oviduct. The most crucial step for initial infection is the adhesion of *M. gallisepticum* to the host cell surface which is achieved by cytoadherence proteins GapA and cytoadherence-related molecule A (CrmA) (5). Previous reports suggest that the absence of GapA and CrmA in mutant strains of *M. gallisepticum* does not cause infection, proving the importance of these two cytoadherence proteins in infection. Further, the fibronectin-binding proteins M. pneumoniae-like protein A (PlpA) and HMW3-like protein (Hlp3) are known as cytoadherence accessory proteins because they are also involved in cytoadherence (6–8); these two protein-encoding genes are absent in avirulent strain *M. gallisepticum* R_high_ (9). After initial interaction with host cells, the variable lipoprotein hemagglutinin A (vlhA) causes extensive phase variation, which makes it possible for *M. gallisepticum* to escape from host immune response (10, 11). The vlhA gene family consists of 43 genes in R low strain, which plays a prominent role in *M. gallisepticum* pathogenesis. Among these, vlhA 1.07, vlhA 4.01, vlhA 2.02, vlhA 3.03, and vlhA 5.13 were predominantly expressed through the course of infection (12, 13). For the development of recombinant vaccine against *M. gallisepticum* infection in chickens, these proteins could be the effective target.

*M. gallisepticum* infection is controlled by antibiotics, which is a regular practice in poultry farms; antibiotics like fluoroquinolones (enrofloxacin, difloxacin), pleuromutilins (tiamulin), tetracyclines, and macrolides (tylosin, tilmicosin) were widely used (14, 15). The extensive usage of antibiotics has paved a way in the emergence and increase of antibiotic resistance bacteria (16). One such example is quinolone resistance in *M. gallisepticum* which happens to be frequent, dampening the treatment and control methods (17). The antibiotics can reduce the population of *M. gallisepticum* in the respiratory tract, thereby reducing the risk of spread to neighboring flocks (18). Currently available vaccines for *M*. *gallisepticum* infections are killed bacterins, live attenuated strains, and recombinant protein; of these, the commercially approved live attenuated vaccine includes 6/85 strain, ts-11, and F strains which are widely used in layers and commercial flocks. Live attenuated vaccines often display pathogenicity as well as side effects, while bacterins are extremely costly and repeated doses are needed to improve the avian immune system. Additionally, vaccination stress and post-vaccination reaction can also affect chickens (19).

Thus, novel recombinant strategies are required for developing more efficient and less expensive vaccines. So far, no *in silico* approaches had been reported for the development of vaccine; however, using *in silico* approaches few virulence genes have been identified for mycoplasma species (8, 20). Moreover, *in silico*-based multi-epitopic vaccines for cancer were tested in animals and showed promising results (21–23). Thus, there is scope for novel *in silico*-based multi-epitopic vaccines to be successful for infectious diseases. Therefore, a successful vaccine is essential to addressing the current state of Mycoplasma infections in chickens.

Immunoinformatics is a rapidly developing field in bioinformatics, which uses up-to-date technologies to predict potential peptides for vaccine development against various diseases. Recent advances in immunoinformatics allow us to exploit the available data to predict the most efficient epitopic regions in antigenic proteins, which further led to epitope-based vaccine development. The B-cells and T-cells play a central role in the activation of immune response against various viral and bacterial infections. Immunization based on epitopic vaccine has high potential in stimulation of humoral and cellular immune response. These vaccines are composed of highly immunogenic B and T cell epitopes which intensify the immune response through B-cell and T-cell activation. The setback of epitopic vaccines is as follows: due to its smaller size, it may not be recognized as an immunogenic substance by the host immune system, but it can be overcome by the usage of multiple epitopes and adjuvant. The prospective advantages of epitope-based peptide vaccine are as follows: it decreases the risk associated with other types of vaccinations; the epitopes can be wisely engineered and optimized to increase its potency in eliciting stronger immune response; and it has chemical stability due to its smaller size. In addition to that, epitope-based vaccines also offer cost effectiveness, safety, and selection-based immunity.

**Figure 1:**
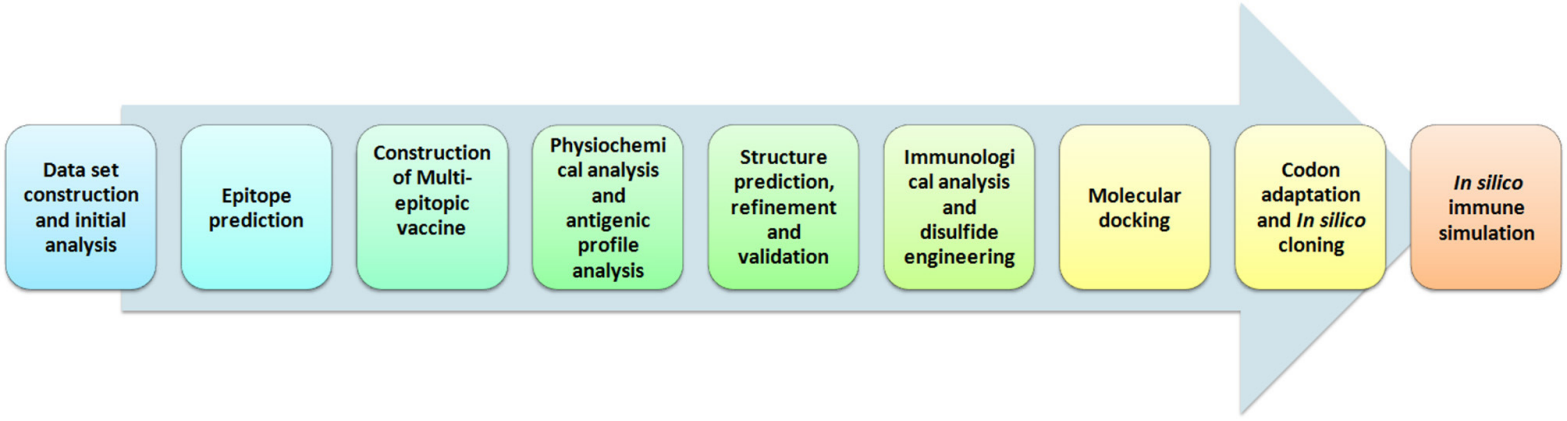
Illustration of steps involved in the development of multi-epitopic vaccine candidate from antigenic protein of *M. gallisepticum*.

Hence, in this study we employed immunoinformatics approaches for the development of epitope-based peptide vaccine from cytoadherence proteins, fibronectin-binding proteins, and variable lipoprotein hemagglutinin A. The selected proteins have a quintessential role in infection and pathogenesis of *M. gallisepticum* into the host cells based upon earlier studies (5, 6, 9, 10, 12, 13). Further, we used reliable computational approaches to predict the highest T-cell as well as B-cell epitopes for these proteins. A multi-epitope vaccine was constructed, validated, and docked against chicken TLR-2 and TLR-5. The pipeline of the computational methods used for vaccine development is depicted in Figure 1. Eventually, this study is aimed at assisting the future laboratory efforts in developing potential vaccine for *M. gallisepticum*. The designed multi-epitopic vaccine could act as a legitimate prophylactic therapy for *M. gallisepticum* and its associated illness in chickens.

## Methodology

### Dataset Construction and Initial Analysis

A dataset was constructed with four cytoadherence proteins and five vlhA proteins of *M. gallisepticum* strain R low from UniProt Knowledgebase. The dataset was further improved by incorporating the protein name and UniProt accession number. Further initial antigenicity and allergenicity analyses for all the proteins were carried out using ANTIGENpro (http://scratch.proteomics.ics.uci.edu/) (24), Vaxign-ML (25), and AllerTOP v. 2.0 (26), respectively.

### Prediction of T Cell Epitope

The lack of chicken MHC alleles in the immunoinformatics database was a major drawback; thus, human MHC alleles were substituted for chicken alleles to predict the epitopes. Previous studies used human alleles to predict epitopes against poultry pathogens (27, 28). Reports have revealed that chicken B–F alleles have the capacity to induce immune response comparable with human class I alleles predominantly during antigen presentation (29, 30). For this reason, the most appropriate human alleles for chicken were chosen from a previous study (31). Alleles HLA^*^B 40:06, HLA^*^B 41:04, and HLA^*^B 41:03 for MHC class I and alleles DRB1:1482, DRB1:1366, DRB1:1310, and DRB1:1445 for MHC class II were chosen.

#### MHC I Binding Epitope Prediction

To predict MHC I binding affinity, the NetMHCcons 1.1 server was used (http://www.cbs.dtu.dk/services/NetMHCcons) (32); the epitopes were screened based on threshold IC50 values: IC50 <50 nM indicates strong binders, whereas IC50 > 500 nM indicates weak binders. The percentage rank for strong binding epitopes was set as 0.5, and that for weak binding epitopes was 2. The length of the epitopes was set as 9 mers. The class I immunogenicity scores for the selected peptides were calculated by the IEDB Analysis Resource (http://tools.iedb.org/immunogenicity) (33).

### MHC II Binding Epitope Prediction

MHC II binding epitopes were predicted using the NetMHCIIpan 3.1 Server (http://www.cbs.dtu.dk/services/NetMHCIIpan-3.1/) (34). Among the available options, the peptide length was set to 15 amino acids. A number of peptides binding to MHC alleles were then predicted by the server, from which peptides were selected based on percentile rank: the percentage rank for strong binding epitopes was set as 0.5, and that for weak binding epitopes was 2.

### B-Cell Epitope Prediction

The potential linear B cell epitopes were predicted by BepiPred (http://tools.iedb.org/bcell/) from the IEDB research resource (35). BepiPred predicts continuous epitopes by combining two residue properties with the hidden Markov model.

### Construction of Multi-Epitopic Vaccine

A multi-epitopic vaccine against *M. gallisepticum* was designed from CTL, HTL, and B cell epitopes from the antigenic proteins. The adjuvant improves the immunogenicity of the vaccine candidate; here, we used the avian β defensin 8, chicken TLR-5 agonist in the N-terminal of the multi-epitopic vaccine construct followed by EAAAK linker and CTL epitopes. The intra-CTL, intra-HTL, and intra-B-cell epitopes were linked using AAY, GPGPG, and KK linkers, respectively.

### Determination of Antigenicity, Allergenicity, and Solubility Profile

ANTIGENpro (http://scratch.proteomics.ics.uci.edu/) (24) predicted the antigenicity of the multi-epitope vaccine, whereas AllerTOP v2.0 (http://www.ddg-pharmfac.net/AllerTOP/) (26) is employed to analyze the allergic nature of the designed vaccine construct. The SOLpro online tool was used for the calculation of the solubility *Escherichia coli* (*E. coli*) host (http://scratch.proteomics.ics.uci.edu/) (36).

### Determination of Physiochemical Properties

The ExPASy-ProtParam server (http://web.expasy.org/protparam/) was employed to calculate the physicochemical properties of the designed multi-epitopic vaccine. This tool computes several parameters like molecular weight, isoelectric point (pI), half-life, atomic composition, aliphatic index, instability index, and grand average hydropathicity (GRAVY) (37).

### Secondary Structure Analysis

A secondary structure analysis of the multi-epitope vaccine construct was performed using the Garnier–Osguthorpe–Robson (GOR IV) online server with mean accuracy of 64.4% (https://npsa-prabi.ibcp.fr/cgi-bin/npsa_automat.pl?page=npsa_gor4.html) (38) and position specific iterated prediction (PSIPRED) analysis on outputs from PSI-BLAST (http://bioinf.cs.ucl.ac.uk/psipred/) (39).

### Tertiary Structure Prediction, Refinement, and Validation

The I-TASSER server was used in tertiary-structure modeling of the vaccine construct (https://zhanglab.ccmb.med.umich.edu/I-TASSER/) (40). The tertiary structure modeled by I-TASSER was refined by the GalaxyRefine server (http://galaxy.seoklab.org/cgi-bin/submit.cgi?type=REFINE) (41). The galaxy server applies molecular dynamic simulation for overall structural relaxation and swaps amino acids with high-probability rotamers to refine the structures. The output generally contains five refined models, with GDT-HA, RMSD, MolProbity, Clash score, Poor rotamers, and Rama favored scores (42). The output was authenticated by PROCHECK (https://servicesn.mbi.ucla.edu/PROCHECK/), which verifies the stereochemical quality of refined protein structures by examining residue by residue and the overall structural geometry (43).

### Immunological Analysis

The presence of linear (continuous) and conformational (discontinuous) B cell epitopes in the multi-epitopic vaccine construct was analyzed. The BcePred online server (https://webs.iiitd.edu.in/raghava/bcepred/index.html) was employed in the prediction of continuous B-cell epitopes (44). ElliPro from IEDB (http://tools.iedb.org/ellipro/) was used in the prediction of discontinuous B-cell epitopes, which predicts epitopes based on a protein's 3D structure (45).

### Multi-Epitope Vaccine Protein Disulfide Engineering

Disulfide bonds offer considerable stability and strengthen the geometric conformation of proteins. The online DbD2 server, available at http://cptweb.cpt.wayne.edu/DbD2/index.php, was used in this purpose. The pair of residues able to form a disulfide bond, if every amino acid residue is mutated to cysteine, is identified by this web server (46).

### Molecular Docking

In this study, interactions between the vaccine construct with chicken TLR-2 and TLR-5 receptors was studied as they are bacterial sensing TLR (47). The active and passive residues in both the receptor and vaccine construct were predicted by CPORT (48). The HADDOCK 2.4 (https://www.bonvinlab.org/software/haddock2.4/) online server performed docking between multi-epitopic vaccine construct and chicken TLR-2 and TLR-5 receptors (49). HADDOCK Refinement Interface was used to refine the best cluster which has the lowest HADDOCK score. The best structure after refinement from the docked complex was chosen, and their binding affinity was calculated using the PRODIGY web server (50, 51). Lastly, PDBsum (https://www.ebi.ac.uk/thorntonsrv/databases/pdbsum/Generate.html) mapped the interacting residues between the docked complexes (52).

### Codon Adaptation and *in silico* Cloning of a Multi-Epitopic Vaccine Construct

The JAVA Codon Adaptation Tool (JCAT) server (http://www.jcat.de) accomplished codon optimization as per *E. coli* codon usage (53). Certain factors that are important to the efficacy of gene expression including codon adaptation index (CAI) and GC content adjustment were enhanced. Cloning and expression of the multi-epitopic vaccine construct in a suitable expression vector were achieved using SnapGene *in silico* cloning.

### *In silico* Immune Simulation

*In silico* immune simulation was performed by the C-ImmSim server (https://kraken.iac.rm.cnr.it/C-IMMSIM/) (54) to analyze the immunogenicity and immune response of the designed multi-epitopic vaccine. The entire simulation ran for 1,100 time steps, where one time step is 8 h. Three peptide injections were given at day 1, day 84, and day 168, and other parameters were kept default.

## Results

### Dataset Construction and Initial Analysis

The dataset for this study was constructed by including the protein name, UniProt accession number, and antigenicity and allergenicity of the selected proteins (Table 1). The threshold score for antigenicity is 0.4; all the proteins obtained scores higher than the threshold score and thus has a potential to induce immune response. All the selected protein sequences are non-allergens.

**Table 1:** Dataset and antigenicity and allergenicity prediction.

**S. no**.	**Protein name**	**UniProt accession number**	**Antigenicity score**	**Allergen**
1.	GapA	Q9REM8	0.961828	Non-allergen
2.	PlpA	Q7NBF9	0.875417	Non-allergen
3.	Hlp3	Q7NBT3	0.777268	Non-allergen
4.	CrmA	F8WJY4	0.935993	Non-allergen
5.	vlhA.1.07	Q7NB51	0.949858	Non-allergen
6.	vlhA.4.01	Q7NBR8	0.940062	Non-allergen
7.	vlhA.2.02	Q7NB24	0.940421	Non-allergen
8.	vlhA.3.03	Q7NAP4	0.960592	Non-allergen
9.	vlhA.5.13	Q7NBD0	0.937955	Non-allergen

### T-Cell Epitope Prediction

Highly ranked MHC-I-binding peptides were selected based on IC50; IC50 <50 nM were identified as strong binders by using the NetMHCcons 1.1 server (Supplementary Table S1). MHC-II binding peptides were predicted using the NetMHCIIpan 3.1 server, and the epitopes with %rank <2 were regarded as strong binders (Supplementary Table S2) and preferred for further studies.

### B-Cell Epitope Prediction

The BepiPred server predicted the B-cell binding epitopes with the threshold score above 0.350 and considered them as probable epitopes. The epitopes from the antigenic proteins of *M. gallisepticum* with length ranging from 10 to 15 peptides are given in Supplementary Table S3.

### Multi-Epitopic Vaccine Design

**Figure 2:**
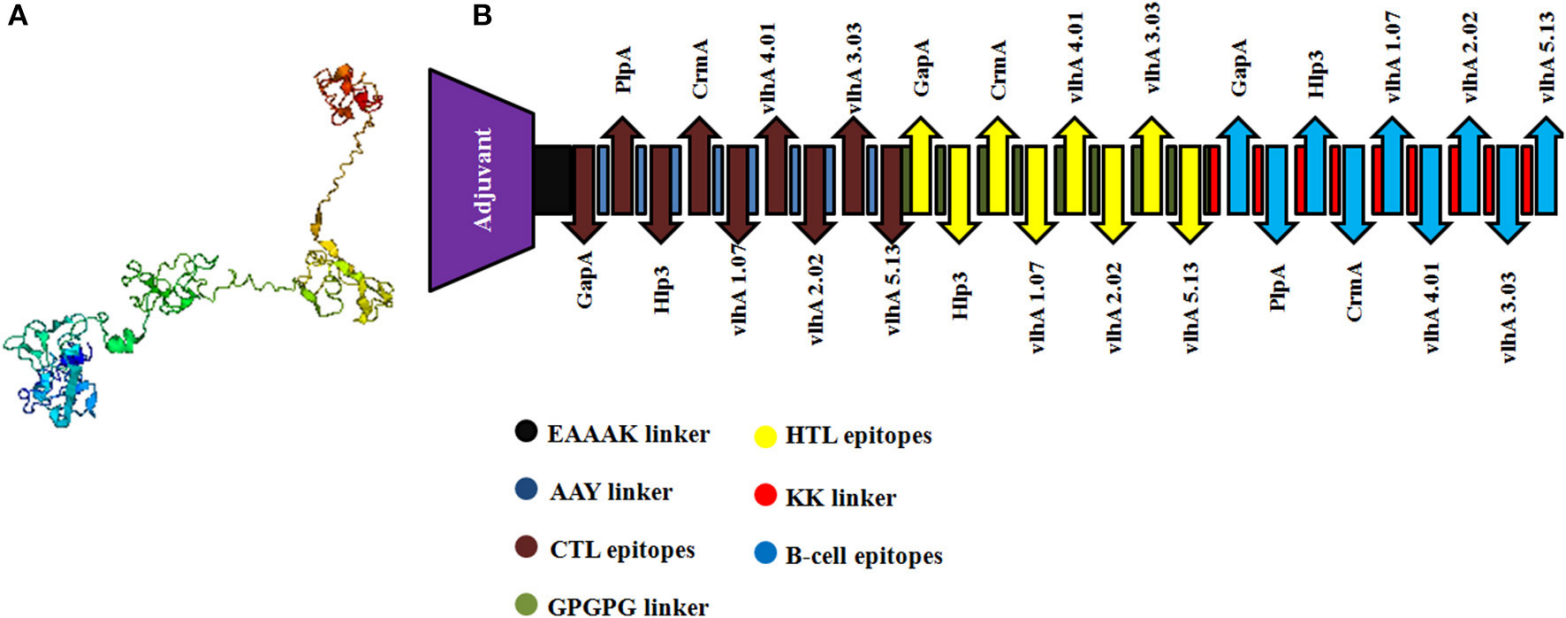
**(A)** Three-dimensional structure of the engineered vaccine construct. **(B)** Graphical representation of the final multi-epitopic vaccine construct along with CTL, HTL, and B-cell epitopes.

The selected MHC-I and MHC-II-binding epitopes and B-cell epitopes derived from *M. gallisepticum* GapA, plpA, Hlp3, CrmA, vlhA 1.07, vlhA 2.02, vlhA 3.03, vlhA 4.01, and vlhA 5.13 were used to design the multi-epitope vaccine construct. Avian β-defensin 8 sequences were added as an adjuvant followed by the epitopes and linked by specific linkers (Figure 2).

### Antigenicity and Allergenicity Prediction of Multi-Epitopic Vaccine

The foremost decisive factor needs to be guaranteed while designing a vaccine, which is the antigenic potential of the vaccine candidate to induce a humoral and/or cell-mediated immune response against the targeted virus and allergenicity of the constructed vaccine. The antigenicity score for multi-epitope vaccine was 0.683753 as predicted by the ANTIGENpro server. The final vaccine construct was predicted to be non-allergenic by AllerTOP v2.0. The solubility prediction using the SOLpro server illustrated that the vaccine construct is soluble with a probability of 0.749662.

### Physiochemical Characterization of the Designed Vaccine

The constructed multi-epitope vaccine contains 498 amino acids and a molecular weight of ~54.15 kDa. The estimated pI was 9.75, which points out the alkaline nature of the multi-epitopic vaccine. The estimated instability index, aliphatic index, and grand average of hydropathicity (GRAVY) were 26.09, 79.64, and −0.386, respectively, suggesting that the vaccine is stable, thermostable, and hydrophilic in nature. The total number of positively charged residues (Asp+ Glu) was predicted to be 65 while the total number of negatively charged residues (Arg+ Lys) was predicted to be 33. The extinction coefficient at 280 nm in H_2_O was 47,595. The estimated half-lives in mammalian reticulocytes (*in vitro*), yeast cells (*in vivo*), and *E. coli* (*in vivo*) were 30 h, <20 h, and <10 h, respectively.

### Secondary and Tertiary Structure Analysis

**Figure 3:**
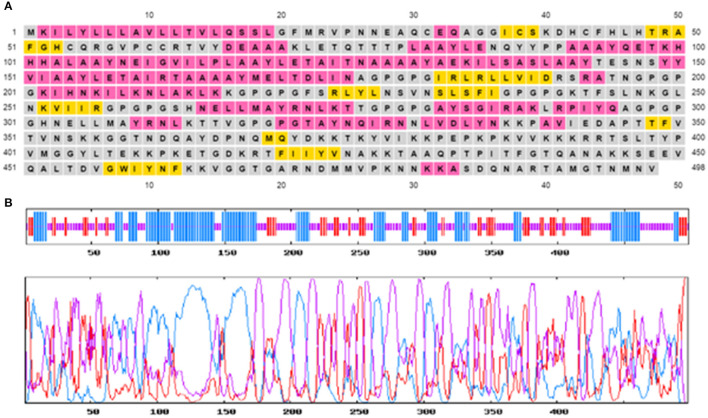
Predicted secondary structure of the multi-epitope vaccine construct. **(A)** Graphical illustration of the secondary structure by the PSIPRED server. **(B)** GOR IV secondary structure prediction-alpha helix (blue), random coils (yellow), and extended strand (red).

The secondary structure of the multi-epitopic vaccine consists of 48.80% (243 aa) of random coils, 34.74% (173 aa) alpha-helices, and 16.47% (82 aa) betasheets. Figure 3 represents the graphical image of the GOR IV server and PSIPRED server. The tertiary structure of the vaccine was modeled using the I-TASSER server. The I-TASSER server modeled five tertiary structures of the designed multi-epitopic vaccine protein based on 10 threading templates. All the 10 preferred templates showed high-quality alignment as per their Z-score values (ranging from 0.38 to 2.40). The predicted model has a C-score value of −2.31. The preferred model had an estimated TM score of 0.44 ± 0.14 and RMSD of 12.9 ± 4.2Å. The TM score indicates the structural similarity between two structures. A TM score >0.5 specifies a model of correct topology, and a TM score <0.17 means random similarity.

### Multi-Epitopic Vaccine Structure Refinement and Validation

**Figure 4:**
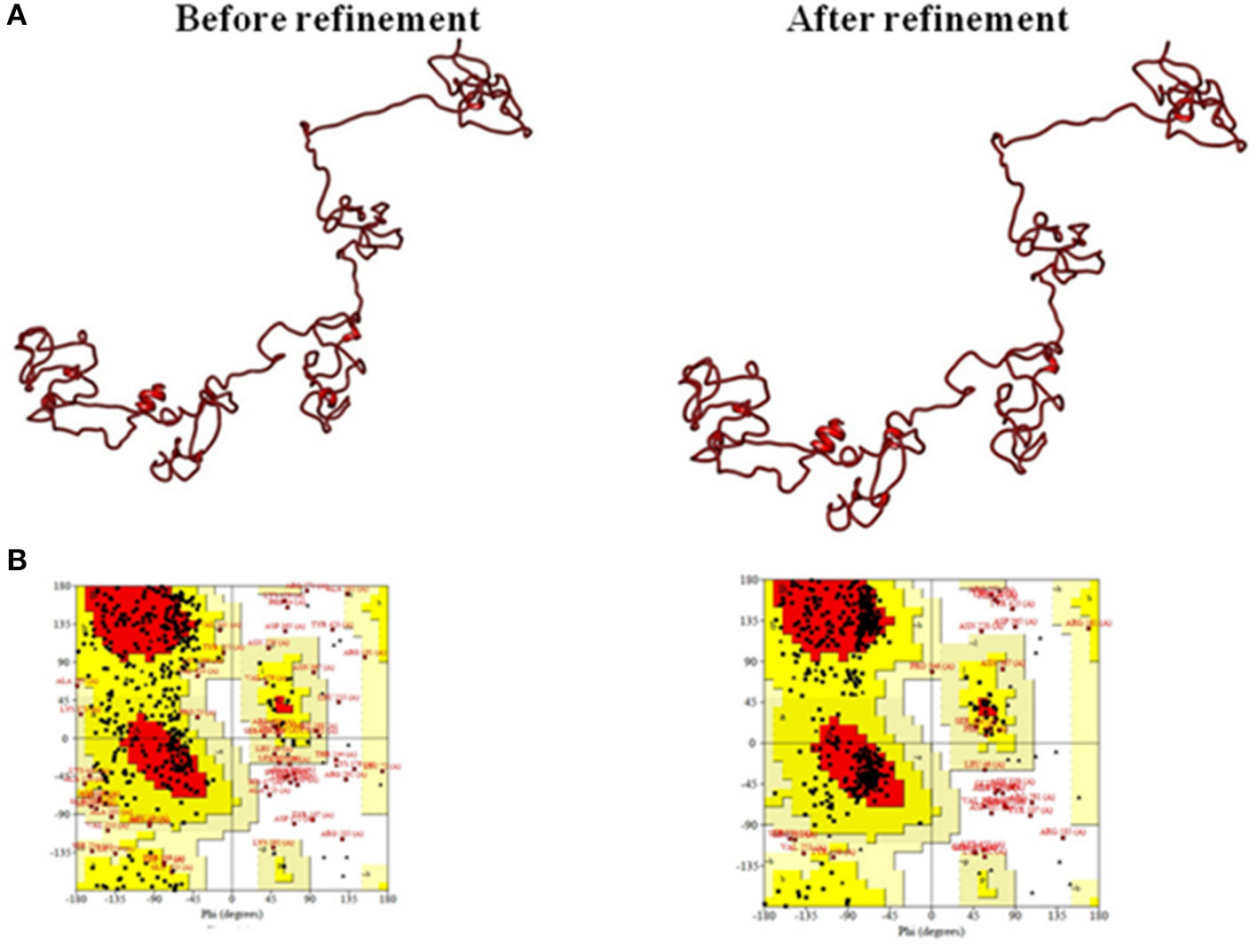
**(A)** Tertiary structure model of the multi-epitope vaccine construct and **(B)** Ramachandran plot validation.

The 3D structure modeled by the I-TASSER server was validated by the PROCHECK server and Ramachandran plot analysis was performed, and the output revealed that 52.6% of residues fall in the favored region. For further refinement, the GalaxyRefine server (http://galaxy.seoklab.org/cgi-bin/submit.cgi?type=REFINE) was used; the output provided five refined models for the multi-epitopic vaccine construct (Supplementary Table S4). Among them, model four was considered to be the best-refined structure with parameters such as GDT-HA of 0.8886, RMSD of 0.576, MolProbity of 2.565, Clash score of 19.2, Poor rotamers of 1.2, and favored Rama of 81.7%. Thus, this model (Figure 4) was finally selected for further investigations.

### Immunological Property Assessment

**Figure 5:**
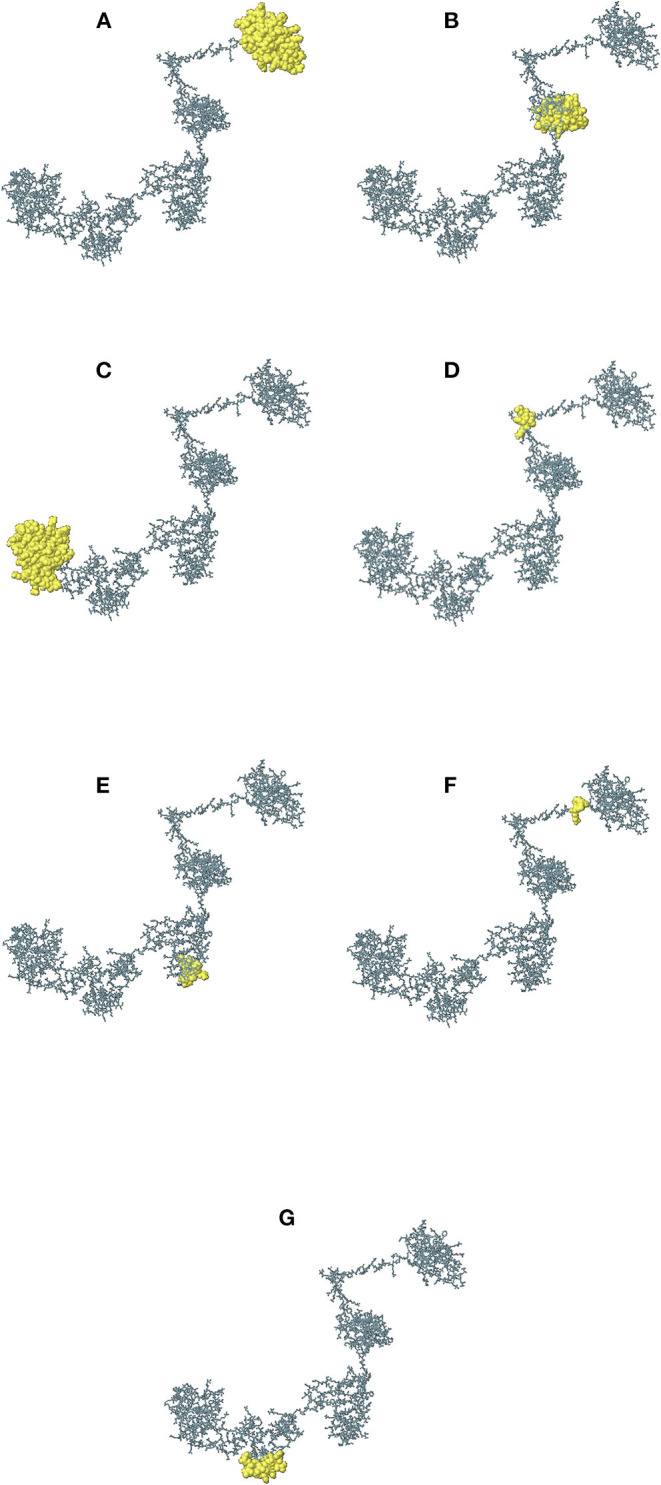
The immunogenic conformational B-cell epitopes are represented as yellow globules. The details of **(A–G)** are given in Supplementary Table S5.

The continuous B-cell epitopes in multi-epitope vaccine was predicted by BcePred with default parameters. Vital properties of epitopes like hydrophilicity, antigenicity, flexibility, accessibility, polarity, and exposed surfaces were predicted (Table 2). Conformational B cell epitopes in multi-epitope vaccine were predicted by ElliPro. In total, seven conformational epitopes were predicted; the details of the prediction are given in Supplementary Table S5 and Figure 5.

**Table 2:** Results of linear B-cell epitopes in multi-epitope vaccine by the BcePred server.

**Prediction parameters**	**Epitope position**
Hydrophilicity	28–31, 32, 76, 145, 193–196, 198–199, 218, 240, 259, 262, 277–278, 301, 354–365, 373, 393, 410–416, 442–449, 469–470, 480–488
Flexibility	190–192, 197, 213, 215–216, 237–238, 247, 256–257, 274, 351–357, 380–382, 389–392, 407–414, 443–445, 463, 477–482, 484
Accessibility	25–30, 65, 68, 73–77, 85–90, 96–100, 145–148, 192–195, 203, 207, 212–213, 215–218, 245, 249–250, 271–275, 277, 289–290, 292, 310–313, 323, 325–328, 335–338, 353–354, 356–359, 361–397, 407–417, 426– 431, 442–449, 461, 478– 488
Exposed surface	98, 212–213, 215–216, 332–337, 370–394, 408–416, 443–448, 478–484,
Polarity	42–48, 50, 97–103, 203–207, 212–213, 288–290, 373–375, 380–385, 388–395, 407– 416, 445–449, 479–481
Antigenic propensity	4–5, 9, 13–17, 40–41, 43–46, 56–63, 112–113, 149, 185–189, 227–228, 233, 334, 349, 386–387, 398–399, 421–422

*The epitopes were listed based on hydrophilicity, flexibility, accessibility, exposed surface, polarity, and antigenic propensity*.

### Vaccine Protein Disulfide Engineering

**Figure 6:**
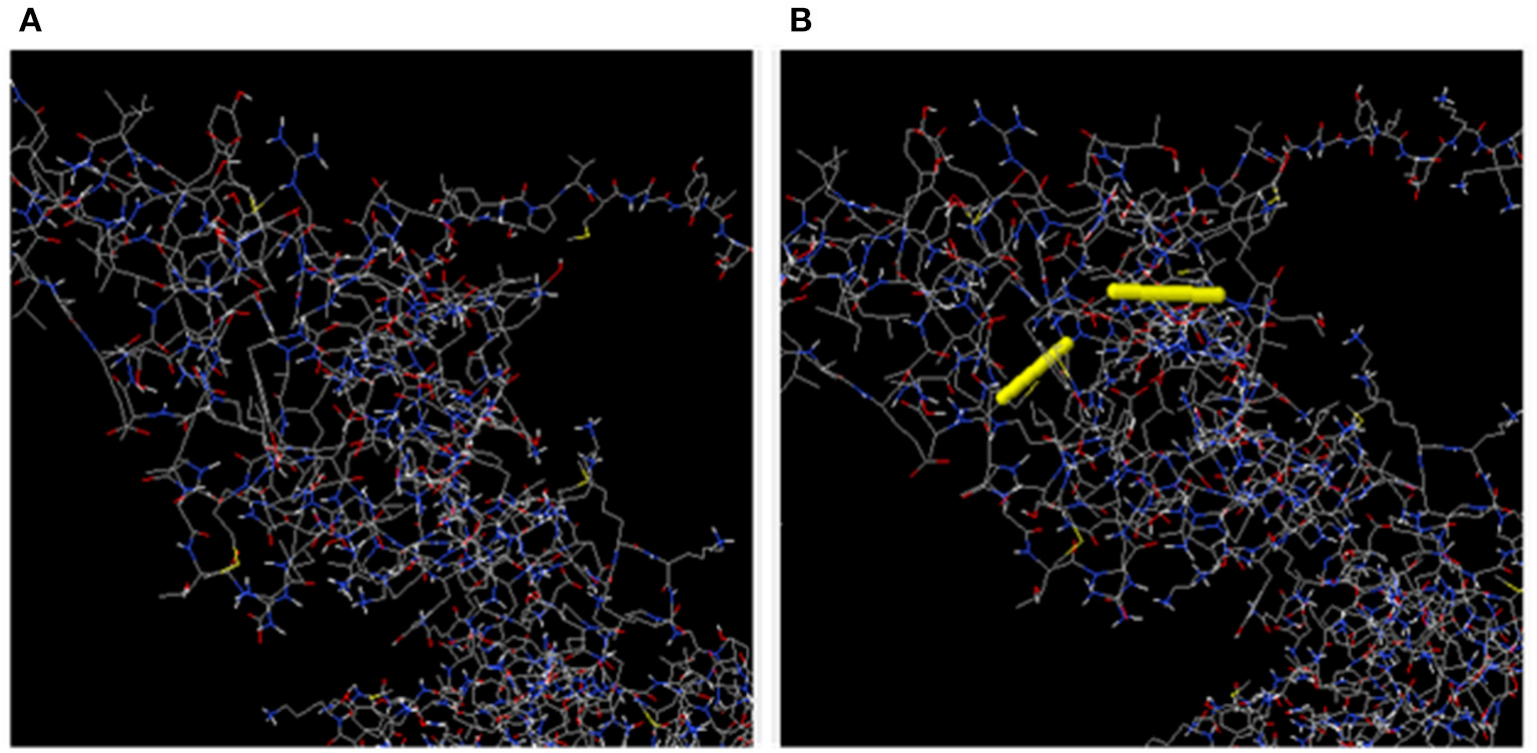
**(A)** Initial model without disulphide bonds. **(B)** Mutant model; the yellow stick represents the disulphide bond formation.

In total, 55 pairs of amino acid residues were predicted to form a disulfide bond using the DbD2 server. Just two residues, including GLU 145-SER 148 and ILE 152-ALA 156 were substituted by cysteine residues and found to fulfill the disulfide bond formation with residue assessment by chi3 and B-factor energy parameters (Figure 6). The residue screening was performed based on chi3 value (−87 to +97) and <2.5 energy value.

### Molecular Docking

**Figure 7:**
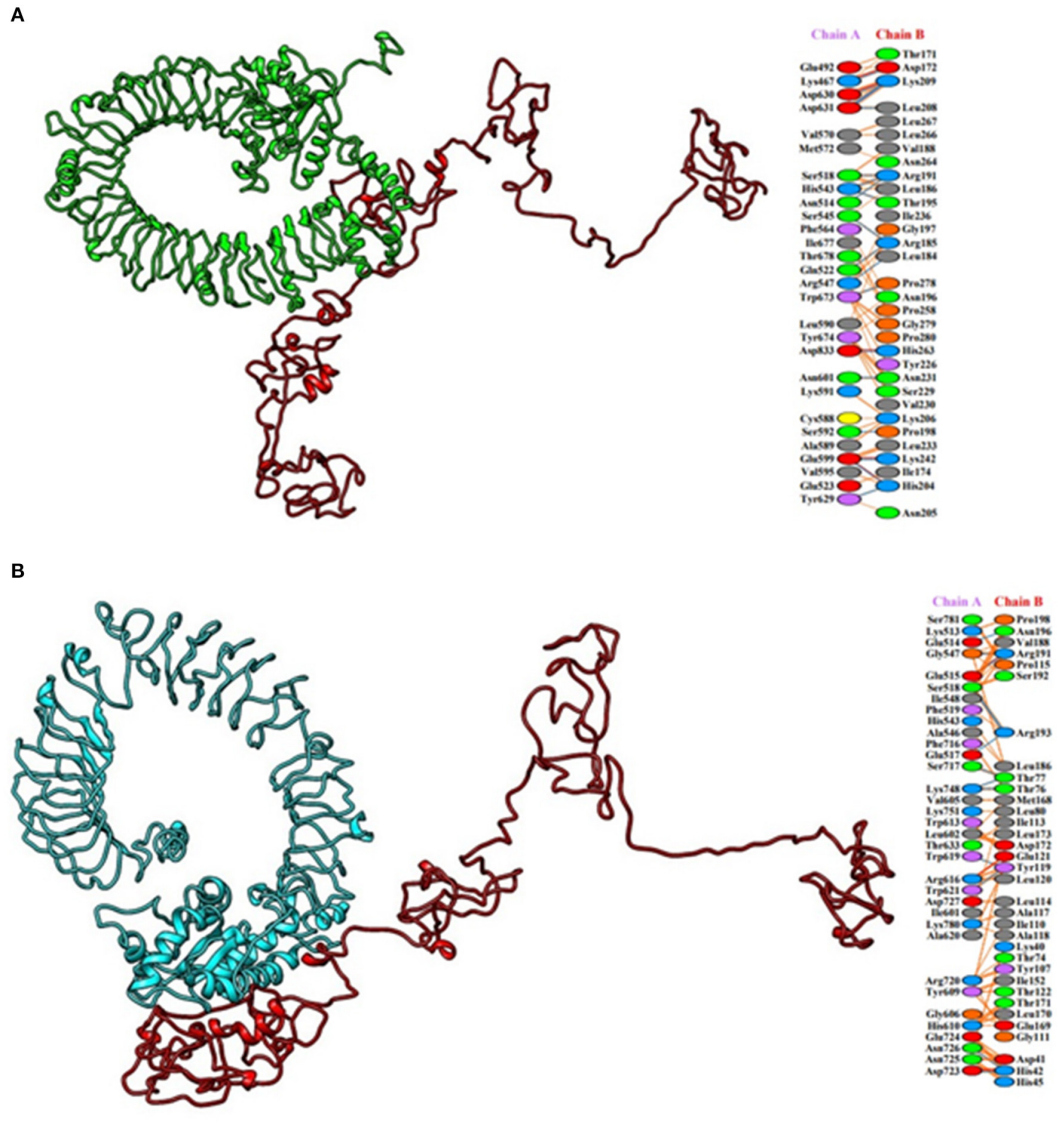
Molecular docking of multi-epitopic vaccine with chicken immune receptors. **(A)** Chicken TLR-2 (green) and multi-epitopic vaccine (red). **(B)** Chicken TLR-5 (cyan) and multi-epitopic vaccine (red).

HADDOCK clustered 133 structures in 15 clusters, showing 66% of the models formed by water-refined HADDOCK for multi-epitopic vaccine-Chicken TLR-2 complex and 78 structures in 11 clusters, showing 39% of the models formed by water-refined HADDOCK for multi-epitopic vaccine-Chicken TLR-5 complex. The most precise predicted cluster is the cluster with the smallest HADDOCK ranking. The HADDOCK refinement server refined the top cluster where 20 structures were grouped into one cluster, resulting in 100% of the HADDOCK water-refined version for both the complexes. In Table 3, the results of the refined models were presented. A strong binding affinity between the multi-epitopic vaccine and chicken TLR-2 receptor and chicken TLR-5 receptor is indicated by the Haddock scores of −225.0 ± 4.2 and −230.9 ± 0.0, respectively; a negative score suggests better docking. The docked complex along with interacting residues between docked complexes is shown in Figure 7. A total of 19 hydrogen bonds, 6 salt bridges, and 160 non-bonded contacts were analyzed between the multi-epitopic vaccine and chicken TLR-2 receptor, and 13 hydrogen bonds, 3 salt bridges, and 150 non-bonded contacts were analyzed between the multi-epitopic vaccine and the chicken TLR-5 receptor. The Gibbs-free energy (ΔG) is a decisive parameter for deciding that the probability of an interaction can occur under specific conditions in the cell. The binding affinity of the docked complex was analyzed by the PRODIGY web server. The G-values were −14.4 and −14.6 kcal mol-1, and the dissociation constant was 2.9 E-11 and 2.0 E-11 for the vaccine construct and chicken TLR-2 and TLR-5, respectively. The results indicated by negative Gibbs-free energy (ΔG) shows that the docked complex is energetically viable.

**Table 3:** Docking statistics of best-refined docked chicken TLR-2, TLR-5, and vaccine construct.

**Parameters**	**Chicken TLR-2- multiepitopic vaccine**	**Chicken TLR-5-multiepitopic vaccine**
HADDOCK score	−225.0 ± 4.2	−230.9 ± 0.0
Cluster size	20	20
RMSD from the overall lowest energy structure	0.7 ± 0.4	0.0 ± 0.0
Van der Waals energy	−106.6 ± 2.3	−137.7 ± 0.0
Electrostatic energy	−470.8 ± 21.7	−330.2 ± 0.0
Desolvation energy	−24.3 ± 2.5	−27.2 ± 0.0
Restraints violation energy	0.0 ± 0.0	0.0 ± 0.0
Buried surface area	3581.5 ± 62.8	4029.3 ± 0.0

### Codon Adaptation and *in silico* Cloning

**Figure 8:**
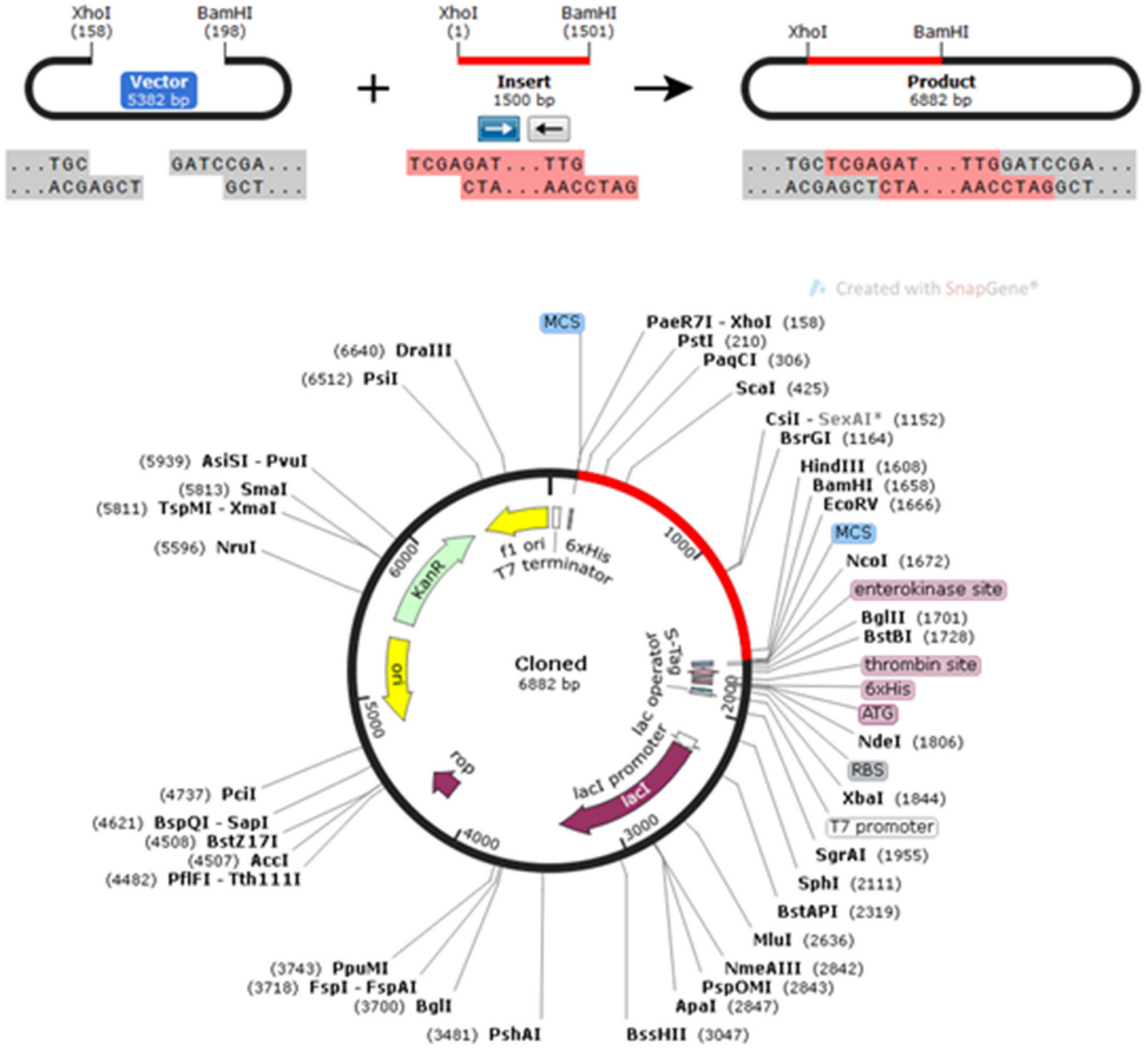
*In silico* restriction cloning of the gene sequence of the vaccine construct into the pET30a(+) expression vector; the red-colored region represents the gene coding the multi-epitopic vaccine, and the black-colored region points out the vector backbone.

The CAI in the adapted sequence was 0.9713, and the GC content of the enhanced sequence was expected to be 51.74%, whereas the GC content of *E. coli* was 50.7. To perform *in silico* cloning, the vaccine construct sequence was examined for restriction enzyme sites; the Xho I and Bam HI restriction enzyme sites were not present in the vaccine sequence, so these enzymes were used for *in silico* cloning in the pET30a (+) vector. A clone of 6,882 bp was achieved subsequent to introduction of the vaccine construct into the pET30a (+) vector (Figure 8).

### *In silico* Immune Simulation

**Figure 9:**
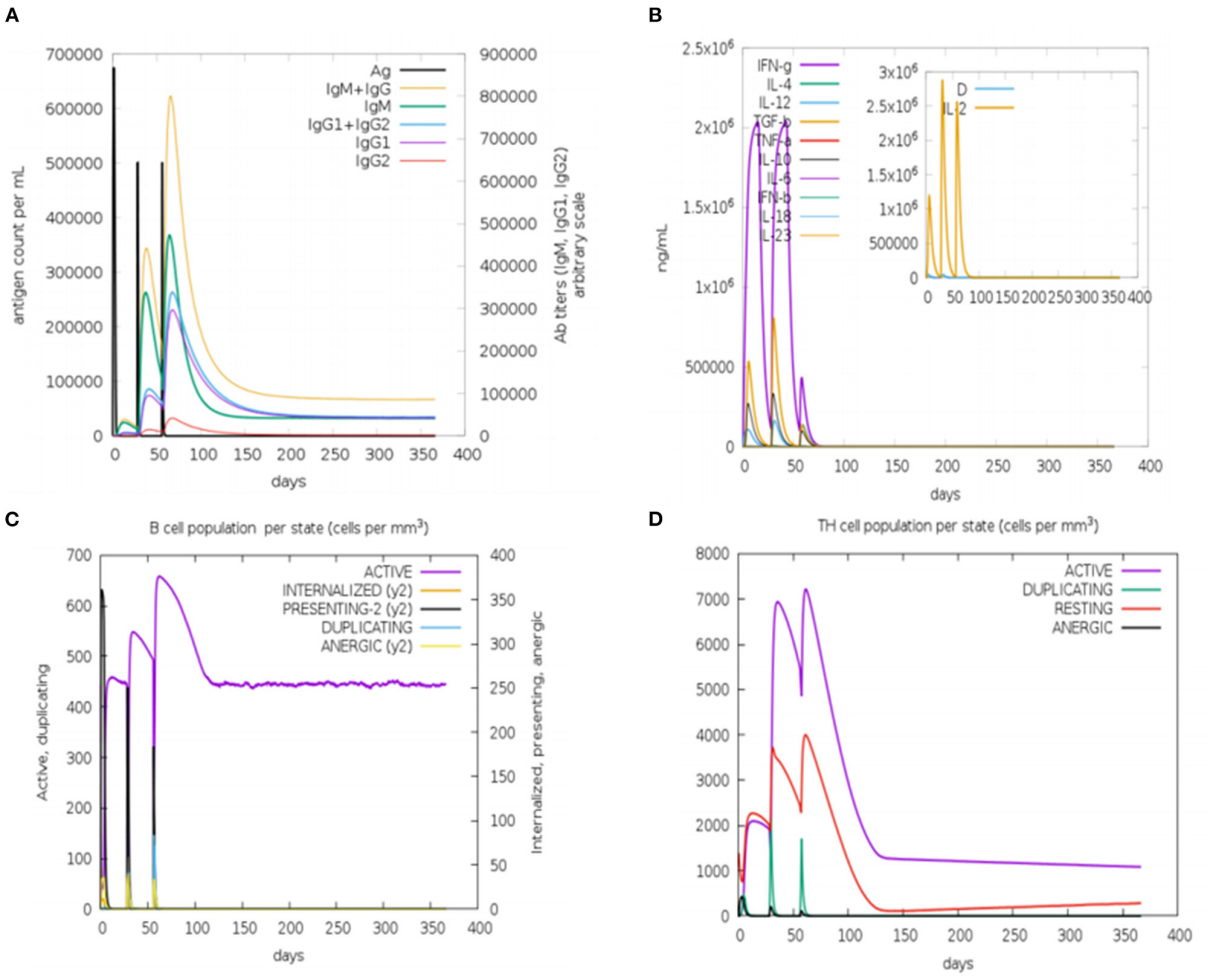
*In silico* immune simulation of multi-epitopic vaccine by the C-ImmSim server. **(A)** Antibody production in response to antigen injections (vertical black lines). **(B)** Expression of cytokine level. **(C)** Level of B-cell population. **(D)** Level of T-cell population.

*In silico* immune simulation was performed to characterize the immune profile of the designed multi-epitopic vaccine construct. The immune response was extensively activated, and there is an increase in antibody titer after injection Figure 9A. The cytokine response was also analyzed; the results show reliable and vigorous response following injection (Figure 9B). The B and T cell populations were also increased considerably (Figures 9C,D). These results confirm that the designed multi-epitopic vaccine effectively elicits an immune response.

## Discussion

Vaccines are significantly accountable for providing host organisms with defense from a specific disease. The conventional vaccine development process is extremely meticulous, expensive, and time consuming. Immunoinformatics tools are a boon to design and develop vaccine in a short period of time with specificity and ease. It allows detecting pathogens underlying immunogenic proteins, together with prediction of diverse immune-dominant epitopes which are involved in the development of humoral and cell-mediated immune responses against the pathogen. For that reason, a multi-epitope-based peptide vaccine can be designed with immunogenic proteins of a pathogen. In the present scenario, a large number of peptide vaccines are in progress, majority of which are for human infectious diseases and tumor (55–58). Very few studies are reported in the field of *in silico* vaccine for poultry and animals. Previous reports focused on the prediction of epitopes for animal diseases like foot and mouth disease (59) and animal trypanosomiasis (60) in designing the multi-epitopic vaccine and offered effective immunity as evaluated with currently available vaccines. The following studies validate the immunoinformatics method to design multi-epitopic vaccines against infectious diseases in poultry (61–64). The advantages of this method include evaluation of complete antigenic epitopes and molecular modeling to study the probable binding with host proteins (65, 66). The *in silico* validations like molecular docking and *in silico* cloning and immune simulation (67–70) were included in this study to make the constructed multi-epitope as functionally suitable as a vaccine candidate against *M. gallisepticum* infection.

The purpose of multi-epitopic vaccine is to identify the specific CTL, HTL, and B-cell epitopes from the antigenic proteins that induce specific and effective immune response. Initial antigenicity and allergenicity analyses revealed that selected proteins are antigenic and non-allergic. Vaccine design requires an adjuvant that is necessary for increasing the effectiveness of the vaccine (71); hence, avian β-defensin was used as an adjuvant at the N-terminal followed by a sequence of various CTL, HTL, and B-cell epitopes present in antigenic proteins (72). The selected epitopes together with the adjuvant have been merged together with the help of suitable linkers. A good vaccine candidate is supposed to be competent in initiating an immune response without causing an allergic reaction; hence, the antigenicity and allergenicity of the multi-epitopic vaccine construct were analyzed. The vaccine construct is antigenic and non-allergic in nature. The molecular weight was 54.15 kDa, and the theoretical pI was 9.75, which signifies the basic nature of the vaccine construct. The aliphatic index and GRAVY score indicate that the vaccine is thermostable and hydrophilic. The secondary structure of the vaccine construct revealed that the random coil dominated the structure followed by alpha-helices and beta sheets. The tertiary structure was selected based on the highest c-score. The structure was further refined, and a better structure was obtained after refinement. The prediction of discontinuous and continuous B-cell epitopes in the vaccine construct shows that it can possibly interact with antibodies and is flexible. Further disulfide engineering was performed to stabilize the vaccine construct. The interaction prototype of the vaccine construct with chicken TLR-2 and TLR-5 receptors was analyzed by molecular docking studies. The results indicated a better interaction. The negative Gibbs-free energy (ΔG) indicates that the docked complex is energetically viable. Codon optimization was done for the *E. coli* K12 strain; the estimated GC content and CAI indicate enhanced the transcriptional and translational efficiency. Restriction enzymes Xho I and Bam HI were selected for cloning in the pET30a(+)vector. The expression of codon was adequate. In addition to this, *in silico* immune simulation results revealed that upon injection of the designed multi-epitopic vaccine, a strong immune response was triggered.

The currently available live attenuated vaccine and bacterins are commonly used in commercial birds; these vaccines cannot aid in control during the sudden onset of *M. gallisepticum* infection; strict biosecurity has to be followed to control and eradicate the infection. The live attenuated vaccine shows adverse side effects and pathogenicity, while the bacterins are associated with high cost and repeated dosage. The limitations of the currently available vaccine can be addressed by the present study which employs robust immunoinformatics tools to design multi-epitopic vaccine, and the result demonstrates that the designed multi-epitopic vaccine construct have every aspect be developed as an effective vaccine candidate, such as immune specificity, small size, and absence of adverse effects.

## Conclusion

The current study listed out the applications of immunoinformatics to predict the epitopes of *M. gallisepticum* as a potential strategy to speed up vaccine development. *M. gallisepticum* infection has emerged as a severe problem in the poultry industry, resulting in huge economic loss. The present treatment strategies could not effectively eradicate the *M. gallisepticum* infection. Recently, there have been numerous attempts in developing vaccines against *M. gallisepticum*, which are mostly limited to live attenuated vaccines. This study predicted the potential CTL, HTL, and B-cell epitopes from the antigenic proteins against *M. gallisepticum* and designed a multi-epitope vaccine candidate. This approach of vaccine development will have advantages over the classical vaccine development methods by using small epitopes that it will bind specifically to the paratopic region of the host immune cell and elicit higher immune response. The results reported in this study could be supported by *in vivo* and *in vitro* laboratory evaluations in the future. Nevertheless, these findings provide a novel way to develop vaccine against *M. gallisepticum*.

## Data Availability Statement

The original contributions presented in the study are included in the article/Supplementary Material, further inquiries can be directed to the corresponding author/s.

## Author Contributions

SPM and HMC designed and performed the experimental studies. SPM carried out the *in silico* experiments. The manuscript was written by SPM and HMC.

## Conflict of Interest

The authors declare that the research was conducted in the absence of any commercial or financial relationships that could be construed as a potential conflict of interest.

## Publisher's Note

All claims expressed in this article are solely those of the authors and do not necessarily represent those of their affiliated organizations, or those of the publisher, the editors and the reviewers. Any product that may be evaluated in this article, or claim that may be made by its manufacturer, is not guaranteed or endorsed by the publisher.
