# Supplementary material for: A Computational Reverse Vaccinology Approach for the Design and Development of Multi-Epitopic Vaccine Against Avian Pathogen Mycoplasma gallisepticum
Source: Front Vet Sci. 2021 Oct 26;8:721061. doi: 10.3389/fvets.2021.721061 (PMC8577832; doi:10.3389/fvets.2021.721061)
Supplement: Supplementary file 1 [file Data_Sheet_1.docx]

**Supplementary Table 1**: List of predicted MHCI binding epitopes

| **S.No** | **Protein** | **Position** | **Allele** | **Peptide** | **1-log50k(aff)** | **Affinity(nM)** | **%Rank** | **Class I Immunogenicity score** | **Vaxign-ML scores** |
| --- | --- | --- | --- | --- | --- | --- | --- | --- | --- |
| 1. | GapA | 984 | HLA-B40:06 | LETQTTTPL | 0.432 | 569.34 | 0.20 | 0.03484 | 99.3 |
|  |  |  | HLA-B41:04 |  | 0.530 | 162.13 | 0.40 |  |  |
|  |  |  | HLA-B41:03 |  | 0.672 | 34.77 | 0.10 |  |  |
| 2. | PlpA | 817 | HLA-B40:06 | LENQYYPPA | 0.516 | 188.31 | 0.50 | 0.14158 | 90.9 |
|  |  |  | HLA-B41:04 |  | 0.516 | 187.77 | 0.50 |  |  |
| 3. | Hlp3 | 907 | HLA-B41:03 | QETKHHHAL | 0.663 | 38.54 | 0.12 | 0.05082 | 95.3 |
|  |  |  | HLA-B41:04 |  | 0.560 | 117.14 | 0.25 |  |  |
| 4. | CrmA | 769 | HLA-B40:06 | NEIGVILPL | 0.518 | 184.51 | 0.12 | 0.22694 | 97.3 |
|  |  |  | HLA-B41:03 |  | 0.693 | 27.86 | 0.05 |  |  |
|  |  |  | HLA-B41:04 |  | 0.594 | 80.45 | 0.15 |  |  |
| 5. | vlhA.1.07 | 307 | HLA-B40:06 | LETAITNAA | 0.421 | 523.67 | 0.50 | 0.23551 | 100 |
| 6. | vlhA.4.01 | 170 | HLA-B41:03 | AEKILSASL | 0.679 | 32.24 | 0.07 | -0.16625 | 99.6 |
|  |  |  | HLA-B41:04 |  | 0.691 | 28.16 | 0.05 |  |  |
| 7. | vlhA.2.02 | 446 | HLA-B41:03 | TESNSYYVI | 0.642 | 48.11 | 0.20 | -0.20379 | 98.5 |
|  |  |  | HLA-B41:04 |  | 0.523 | 173.85 | 0.40 |  |  |
| 8. | vlhA.3.03 | 119 | HLA-B40:06 | LETAIRTAA | 0.424 | 510.63 | 0.40 | 0.28591 | 99.7 |
| 9. | vlhA 5.13 | 48 | HLA-B40:06 | MELTDLINA | 0.405 | 626.08 | 0.50 | 0.15516 | 99.1 |

**Supplementary Table 2:** MHC-II binding epitopes.

| **S.No** | **Protein** | **Position** | **Allele** | **Peptide** | **Core peptide** | **1-log50k(aff)** | **Binding Affinity(nM)** | **%Rank** | **Vaxign-ML scores** |
| --- | --- | --- | --- | --- | --- | --- | --- | --- | --- |
| 1. | GapA | 941 | DRB1_1482 | IRLRLLVIDRSRATN | LLVIDRSRA | 0.660 | 39.48 | 0.09 | 99.3 |
|  |  |  | DRB1_1445 |  |  | 0.560 | 117.07 | 0.12 |  |
|  |  |  | DRB1_1366 |  |  | 0.780 | 10.84 | 0.40 |  |
|  |  |  | DRB1_1310 |  |  | 0.731 | 18.29 | 0.70 |  |
| 2. | Hlp3 | 5 | DRB1_1482 | KIHNKILKNLAKLKK | ILKNLAKLK | 0.601 | 75.28 | 0.60 | 95.3 |
|  |  |  | DRB1_1445 |  |  | 0.523 | 173.55 | 0.40 |  |
|  |  |  | DRB1_1366 |  |  | 0.782 | 10.61 | 0.40 |  |
|  |  |  | DRB1_1310 |  |  | 0.728 | 19.06 | 0.80 |  |
| 3. | CrmA | 356 | DRB1_1445 | FSRLYLNSVNSLSFI | LYLNSVNSL | 0.477 | 286.75 | 1.40 | 97.3 |
|  |  |  | DRB1_1366 |  |  | 0.743 | 16.09 | 1.40 |  |
|  |  |  | DRB1_1310 |  |  | 0.730 | 18.57 | 0.70 |  |
| 4. | VlhA 1.07 | 690 | DRB1_1445 | KTFSLNKGLNKVIIR | FTLSTSMPA | 0.467 | 320.42 | 1.80 | 100 |
|  |  | 666 | DRB1_1310 | KTFSLNKGLNKVIIR | LNKGLNKVI | 0.700 | 25.58 | 1.60 |  |
| 5. | VlhA 4.01 | 124 | DRB1_1482 | SHNELLMAYRNLKTT | LMAYRNLKT | 0.605 | 71.48 | 0.60 | 99.6 |
|  |  |  | DRB1_1445 |  |  | 0.487 | 256.00 | 1.10 |  |
|  |  |  | DRB1_1366 |  |  | 0.763 | 12.94 | 0.80 |  |
|  |  |  | DRB1_1310 |  |  | 0.695 | 27.13 | 1.80 |  |
| 6. | VlhA 2.02 | 161 | DRB1_1482 | AYSGIRAKLRPIYQA | IRAKLRPIY | 0.626 | 57.19 | 0.30 | 98.5 |
|  |  |  | DRB1_1445 |  |  | 0.510 | 201.61 | 0.60 |  |
|  |  |  | DRB1_1366 |  |  | 0.756 | 14.4 | 1.00 |  |
|  |  |  | DRB1_1310 |  |  | 0.716 | 21.69 | 1.10 |  |
| 7. | VlhA 3.03 | 112 | DRB1_1482 | HNELLMAYRNLKTTV | LMAYRNLKT | 0.625 | 57.81 | 0.30 | 99.7 |
|  |  |  | DRB1_1445 |  |  | 0.506 | 210.38 | 0.70 |  |
|  |  |  | DRB1_1366 |  |  | 0.781 | 10.72 | 0.40 |  |
|  |  |  | DRB1_1310 |  |  | 0.718 | 21.06 | 1.00 |  |
| 8. | VlhA 5.13 | 147 | DRB1_1482 | TAYNQIRNNLVDLYN | YNQIRNNLV | 0.578 | 96.38 | 1.10 | 99.1 |
|  |  |  | DRB1_1445 |  |  | 0.487 | 257.36 | 1.10 |  |
|  |  |  | DRB1_1366 |  |  | 0.774 | 11.55 | 0.50 |  |
|  |  |  | DRB1_1310 |  |  | 0.765 | 12.71 | 0.20 |  |

**Supplementary Table 3**: List of predicted B-cell epitopes by ABCpred server.

| Protein | Epitope | Position | Score |
| --- | --- | --- | --- |
| GapA | PAVIEDAPTTFVTVNS | 524 | 0.95 |
|  | GGAITTWPEVQVNYKT | 710 | 0.94 |
|  | GSAITWGTNGGNFLDT | 504 | 0.93 |
| PlPA | GGTNDQAYDPNQMQYD | 139 | 0.95 |
|  | YLVPQPPRQPDYYSNR | 781 | 0.94 |
|  | YEEIQPSFRRRGGRAK | 832 | 0.93 |
| Hlp3 | TKYVIKKPEPKPKVVK | 351 | 0.93 |
|  | TKPVGPKPQPGKKATK | 337 | 0.93 |
|  | HQQPTTRIEREEVVNK | 801 | 0.92 |
| CrmA | RRTSLTYPVMGGYLTE | 588 | 0.95 |
|  | PGSYTAVNTFNQNLSD | 802 | 0.94 |
|  | AGTGNTTNTSQTVSNP | 436 | 0.94 |
| Vlha 1.07 | PKETGDKRTFIIYVNA | 583 | 0.95 |
|  | TGMQTANKTFSLNKGL | 660 | 0.94 |
|  | NGTAPKAIEFKPAATK | 367 | 0.93 |
| Vlha 4. 01 | TAAQPTPITFGTQANA | 358 | 0.96 |
|  | AASCTSTPTPTPTPNP | 23 | 0.95 |
|  | ERTIYNQIFGNTESNE | 425 | 0.91 |
| Vlha 2.02 | SEEVQALTDVGWIYNF | 279 | 0.92 |
|  | GGTSFEGNAPNLGNVT | 562 | 0.89 |
|  | ETVPDDSNRSNNNQDG | 37 | 0.89 |
| Vlha 3.03 | VGGTGARNDMMVPKNN | 495 | 0.93 |
|  | TMNGKTPTVNDINVAK | 377 | 0.91 |
|  | AARMGLTTVFDSKAKN | 63 | 0.90 |
| Vlha 5.13 | ASDQNARTAMGTNMNV | 469 | 0.92 |
|  | NQANTDKTTFDNEHPN | 108 | 0.91 |
|  | SWIYSLAGTNTKYQFS | 282 | 0.90 |

**Supplementary Table 4:** List of models and relevant scores by galaxy refine server.

| **Model** | **GDT-HA** | **RMSD** | **MolProbity** | **Clash score** | **Poor rotamers** | **Rama favored** |
| --- | --- | --- | --- | --- | --- | --- |
| Initial | 1.0000 | 0.000 | 3.406 | 10.6 | 15.5 | 52.6 |
| MODEL 1 | 0.8916 | 0.556 | 2.537 | 19.9 | 1.0 | 79.4 |
| MODEL 2 | 0.8996 | 0.553 | 2.590 | 19.8 | 1.2 | 80.8 |
| MODEL 3 | 0.8901 | 0.563 | 2.584 | 20.1 | 1.2 | 81.7 |
| MODEL 4 | 0.8886 | 0.576 | 2.565 | 19.2 | 1.2 | 81.7 |
| MODEL 5 | 0.8996 | 0.543 | 2.545 | 20.8 | 0.5 | 80.2 |

**Supplementary Table 5:** Conformational B cell epitopes from multi- epitopic vaccine construct.

| **S. NO** | **Epitope position** | **Number of residues** | **Score** |
| --- | --- | --- | --- |
| A | V498, N497, M496, N495, T494, G493, M492, A491, T490, R489, A488, N487, Q486, D485, S484, A483, K482, K481, N480, N479, K478, P477, V476, M475, M474, D473, N472, R471, A470, G469, T468, G467, G466, V465, K464, K463, F462, N461, Y460, I459, W458, G457, V456, D455, T454, L453, A452, Q451, V450, E449, E448, S447, K446, K445, A444, N443, A442, Q441, T440, G439, F438, T437, I436, P435, T434, P433, Q432, A431, A430, T429, K428, K427, A426, N425, V424, Y423, I422, I421, F420, T419, R418, K417, D416, G415, T414,E413 | 86 | 0.84 |
| B | D361, N360, T359, G358, G357, K356, K355, S354, N353, V352, T351, V350, F349, T348, T347, P346, A345, D344, E343, I342, V341, A340, P339, K338, K337, N336, Y335, L334, D333, V332, L331, N330, N329, R328, I327, Q326, N325, Y324, A323, G319, G317, V316, T315, T314, K313, L312, N311, R310, Y309, A308, M307,L306 | 52 | 0.745 |
| C | L114, I113, V112, G111, I110, E109, N108, Y107, A106, A105, L104, A103, H102, H101, K99, T98, E97, Q96, Y95, A94, A93, A92, P91, P90, Y89, Y88, Q87, N86, E85, L84, Y83, A82, A81, L80, P79, T78, T77, T76, Q75, T74, E73, L72, K71, A70, A69, A68, E67, D66, Y65, V64, T63, R62, C61, C60, P59, V58, G57, R56, Q55, C54, H53, G52, F51, A50, R49, T48, H47, L46, F44, C43, H42, K40, S39, C38, I37, G36, G35, A34, Q33, E32, C31, Q30, A29, E28, N27, N26, P25,V24 | 88 | 0.663 |
| D | P400, Y399, T398, L397, S396, T395, K392, K391 | 8 | 0.586 |
| E | Y270, H263, S262, G261, P260, G259, P258, G257, R256, N231, | 10 | 0.564 |
| F | K412, P411, K410 | 3 | 0.56 |
| G | I160, A159, T158, E157, L156, Y155, Y149, S148, N147, S146, E145, T144, Y143, A141, L140 | 15 | 0.511 |
